# NKL homeobox gene activities in hematopoietic stem cells, T-cell development and T-cell leukemia

**DOI:** 10.1371/journal.pone.0171164

**Published:** 2017-02-02

**Authors:** Stefan Nagel, Claudia Pommerenke, Michaela Scherr, Corinna Meyer, Maren Kaufmann, Karin Battmer, Roderick A. F. MacLeod, Hans G. Drexler

**Affiliations:** 1 Department of Human and Animal Cell Lines, Leibniz-Institute DSMZ–German Collection of Microorganisms and Cell Cultures, Braunschweig, Germany; 2 Department of Hematology, Hemostasis, Oncology and Stem Cell Transplantation, Hannover Medical School, Hannover, Germany; Centre for Stem Cell Research, UNITED KINGDOM

## Abstract

T-cell acute lymphoblastic leukemia (T-ALL) cells represent developmentally arrested T-cell progenitors, subsets of which aberrantly express homeobox genes of the NKL subclass, including TLX1, TLX3, NKX2-1, NKX2-5, NKX3-1 and MSX1. Here, we analyzed the transcriptional landscape of all 48 members of the NKL homeobox gene subclass in CD34+ hematopoietic stem and progenitor cells (HSPCs) and during lymphopoiesis, identifying activities of nine particular genes. Four of these were expressed in HSPCs (HHEX, HLX1, NKX2-3 and NKX3-1) and three in common lymphoid progenitors (HHEX, HLX1 and MSX1). Interestingly, our data indicated downregulation of NKL homeobox gene transcripts in late progenitors and mature T-cells, a phenomenon which might explain the oncogenic impact of this group of genes in T-ALL. Using MSX1-expressing T-ALL cell lines as models, we showed that HHEX activates while HLX1, NKX2-3 and NKX3-1 repress MSX1 transcription, demonstrating the mutual regulation and differential activities of these homeobox genes. Analysis of a public T-ALL expression profiling data set comprising 117 patient samples identified 20 aberrantly activated members of the NKL subclass, extending the number of known NKL homeobox oncogene candidates. While 7/20 genes were also active during hematopoiesis, the remaining 13 showed ectopic expression. Finally, comparative analyses of T-ALL patient and cell line profiling data of NKL-positive and NKL-negative samples indicated absence of shared target genes but instead highlighted deregulation of apoptosis as common oncogenic effect. Taken together, we present a comprehensive survey of NKL homeobox genes in early hematopoiesis, T-cell development and T-ALL, showing that these genes generate an NKL-code for the diverse stages of lymphoid development which might be fundamental for regular differentiation.

## Introduction

The source of the human blood system is located in the bone marrow where hematopoietic stem and progenitor cells (HSPC) generate progenitors of both the myeloid and lymphoid lineages. The common lymphoid progenitors (CLP) differentiate into T-cells, B-cells or NK-cells. The subsequent T-cell development begins with the early T-cell progenitors (ETP) which migrate into the thymus where their terminal differentiation proceeds toward mature CD4+ and CD8+ T-cells. Stages of developing thymocytes are classified according to their surface marker expression, whether double negative (DN: CD4-, CD8-), double positive (DP: CD4+, CD8+), or single positive (SP4: CD4+, SP8: CD8+) [1]. Major T-cell signalling pathways include NOTCH, T-cell receptor (TCR), and MAPK. These pathways govern differentiation and selection processes which are important for the generation of functional non-autoimmune T-cells, triggering apoptosis in disqualified cells [1,2]. Bone morphogenic protein (BMP) signalling represents an additional pathway which regulates the development of early T-cells [3,4]. Generally, transcriptional regulation plays a prominent role for T-cell development [5].

T-cell acute lymphoblastic leukemia (T-ALL) is a rare malignancy of the hematopoietic system showing a peak incidence in children. The malignant cells represent developmentally arrested thymocytes which keep on proliferating having escaped apoptotic selection. Major T-ALL oncogenes encode aberrantly expressed transcription factors (TFs) which are normally restricted to early hematopoiesis where they regulate developmental processes, including LMO2, TAL1 and LYL1 [6,7]. Homeobox genes TLX1, TLX3, NKX2-1 and NKX2-5 encode TFs which are physiologically silent during hematopoiesis, but which undergo ectopic activation in transformed thymocytes [8–11]. These genes belong to the NKL subclass of homeobox genes which numbers to date eight aberrantly expressed members in T-ALL, highlighting this group of structurally similar developmental TFs for attention [12,13]. Examples of NKL subclass members which are aberrantly expressed in T-ALL with physiological expression in hematopoietic cells include NKX3-1 and MSX1. These genes are variously (de)regulated via TAL1, LYL1, BMP-pathway, and AUTS2 [14–17].

Homeobox genes play fundamental roles in embryonal development and differentiation processes in the adult. Some represent master genes for specific cell types/tissues like NKX2-5 (heart), NKX2-3 (spleen), or PAX6 (eye) [18–20]. Furthermore, particular homeobox gene families form developmental expression patterns called gene codes. The HOX-code constitutes the anterior-posterior axis of the embryo, while the DLX-code mediates dorso-ventral differentiation of the branchial archs [21–23]. Therefore, aberrant activity of homeobox genes potentially contributes to tumorigenesis in multiple tissues and cell types by inter alia deregulating developmental processes therein [13,24,25].

This study presents a comprehensive analysis of hematopoietic NKL homeobox genes focussing on HSPCs, lymphopoiesis and T-cell development. The specification of a physiological hematopoietic NKL gene expression set enables both the identification of aberrant subclass members and any developmental processes potentially impacted.

## Materials and methods

### Primary cells, cell lines and treatments

Primary samples of CD34+ HSPCs were harvested by leukapheresis from healthy volunteers. The cells were purified to ≥98% CD34+ content by magnetic cell sorting using Clini MACS (Miltenyi Biotech, Bergisch-Gladbach, Germany) and cryopreserved in liquid nitrogen. Approval for this study was obtained from the Ethical Committee of Hannover Medical School, and informed consent was obtained in written form in accordance with the Declaration of Helsinki. T-ALL cell lines are held by the DSMZ (Braunschweig, Germany) and were cultivated as described elsewhere [26]. Gene specific siRNA oligonucleotides and AllStars negative Control siRNA (siControl) were obtained from Qiagen (Hilden, Germany). Expression constructs for HHEX, HLX1, NKX2-3 and NKX3-1 were cloned in vector pCMV6-XL4 and obtained from Origene (Wiesbaden, Germany). SiRNAs (80 pmol) and expression constructs/vector controls (2 μg) were transfected into 1x10^6^ cells by electroporation using the EPI-2500 impulse generator (Fischer, Heidelberg, Germany) at 350 V for 10 ms. Electroporated cells were harvested after 20 h cultivation.

### Expression profiling

Gene expression microarray profiling data were generated using the HG U133 Plus 2.0 gene chip (Affymetrix, High Wycombe, UK). The datasets for primary CD34+ HSPCs and 11 T-ALL cell lines (one sample for each probe, N = 1) were generated at the Institute of Pathology (University of Würzburg, Germany) and generously provided by Prof. Andreas Rosenwald. The expression data are deposited at Gene Expression Omnibus (GEO, www.ncbi.nlm.nih.gov) and available via the numbers GSE87303 and GSE87334, respectively. Expression profiling datasets for B-cell progenitors with N = 5 (GSE12453) [27], mature lymphocytes with N = 3 (GSE72642) [28], and for 117 primary pediatric T-ALL patient samples with N = 1 (GSE26713) [11] were obtained from GEO. The B-cell progenitors have been isolated from peripheral blood by depleting CD27+ subpopulations (T-cells, memory B-cells) and CD11b+ subpopulations (monocytes, macrophages, granulocytes, NK-cells). Subsequently, the remaining cells have been FACS-sorted for the B-cell marker IgD+. Germinal center and plasma cells have been isolated from tonsils according to the following marker expression: CD20high CD38+ CD77+ (centroblasts), CD20high CD38+ CD77- (centrocytes), CD20low CD38high (plasma cells). After RMA-background correction and quantile normalization of the spot intensities, the profiling data were expressed as ratios of the sample mean and subsequently log2 transformed. Data processing was performed via R/Bioconductor using limma and affy packages (http://www.bioconductor.org/). For creation of heat maps we used the software CLUSTER version 2.11 and TREEVIEW version 1.60 (http://rana.lbl.gov/EisenSoftware.htm). To parse biological function of shortlisted genes, gene-annotation enrichment analysis was performed using DAVID bioinformatics resources (http://www.david.ncifcrf.org/) [29]. To compare expression profiling data sets we performed principal component analysis on 30% of the most variable transcripts and hierarchical cluster analysis (Euclidian distance, average linkage).

The public GEO dataset GSE69239 provides RNA sequencing data of several isolated hematopoietic entities with N = 2 [30]. For simplicity we renamed sample Thy1 (CD34+ CD7- CD1a-) as DN1, Thy2 (CD34+ CD7+ CD1a-) as DN2, Thy3 (CD34+ CD7+ CD1a+) as DN3, Thy4 (CD4+ CD8+) as DP, Thy5 (CD4+ CD8-) as SP4, and Thy6 (CD4- CD8+) as SP8. The expression data are given in units of FPKM (fragments per kilobase of mappable gene length and million reads) and were obtained after normalizing the count matrices with sequencing depth and gene effective length [30]. FPKM values which are equal to or exceed 1 were interpreted as positive expression. This procedure was validated by comparative quantification of gene expressions via RQ-PCR of HSPC samples.

### Real-time Quantitative Polymerase Chain-Reaction (RQ-PCR) analyses

Total RNA was extracted from cell line samples using TRIzol reagent (Invitrogen, Darmstadt, Germany). Primary human total RNA used in this study was commercially obtained—isolated from peripheral blood mononuclear cells (PBC), thymus, lymph node (LN), spleen, and bone marrow (BM) from Biochain/BioCat (Heidelberg, Germany), RNA from NK-cells (3H Biomedical, Uppsala, Sweden), and RNA from peripheral CD19-positive B-cells and CD3-positive T-cells from Miltenyi Biotec (Bergisch Gladbach, Germany). cDNA was synthesized from 5 μg RNA by random priming using Superscript II (Invitrogen). RQ-PCR analysis was performed with the 7500 Real-time System, using commercial buffer and primer sets (Applied Biosystems/Life Technologies, Darmstadt, Germany). Quantification of MSX1 was performed as described recently [16]. For normalization of expression levels we analyzed the transcript of TATA box binding protein (TBP). Quantitative analyses were performed twice in triplicate. Standard deviations are presented in the figures as error bars. The statistical significance was assessed by Student´s T-Test and the calculated p-values indicated by asterisks (* p<0.05, ** p<0.01, *** p<0.001, n.s. not significant).

### Protein analyses

Western blots were generated by the semi-dry method. Protein lysate from human spleen was obtained from Origene. Protein lysates from cell lines were prepared using SIGMAFast protease inhibitor cocktail (Sigma, Taufkirchen, Germany). Proteins were transferred onto nitrocellulose membranes (Bio-Rad, München, Germany) and blocked with 5% dry milk powder dissolved in phosphate-buffered-saline buffer (PBS). The following antibodies were used: alpha-Tubulin (Sigma) and NKX2-3 (Abcam, Cambridge, UK). For loading control blots were reversibly stained with Poinceau (Sigma) and detection of alpha-Tubulin (TUBA) was performed thereafter. Secondary antibodies were linked to peroxidase for detection by Western-Lightning-ECL (Perkin Elmer, Waltham, MA, USA). Documentation was performed using the digital system ChemoStar Imager (INTAS, Göttingen, Germany).

### Reporter gene assay

For creation of reporter gene constructs we combined a reporter with a regulatory genomic fragment derived from the upstream region of MSX1, containing a consensus binding site for NKX2-3. We cloned the genomic PCR product of the corresponding upstream region (regulator) and of the HOXA9 gene, comprising exon1-intron1-exon2 (reporter), into the *Hind*III/*Bam*HI and *Eco*RI sites, respectively, of the expression vector pcDNA3 downstream of the CMV enhancer. The oligonucleotides used for the amplification of the MSX1-regulator were obtained from Eurofins MWG (Ebersbach, Germany). Their sequences were as follows: MSX1-for1 5´-CCAAGCTTCAGGCAGATCTTGCATCTCC-3´, MSX1-rev1 5´-ATGGATCCTTATCCTAGGAGAAAGACATACTATTAAC-3´. Introduced restriction sites used for cloning are underlined. Constructs were validated by sequence analysis (Eurofins MWG). Transfections of plasmid-DNA into NIH-3T3 cells were performed using SuperFect Transfection Reagent (Qiagen). Commercial HOXA9 and TBP assays were used for RQ-PCR to quantify the spliced reporter-transcript, corresponding to the regulator activity. A cotransfected commercial luciferase construct served as transfection control and was quantified by the Luciferase Assay System (Promega, Mannheim, Germany) using the luminometer Lumat LB9501 (Berthold Technologies, Bad Wildbad, Germany).

## Results

### Expression of NKL homeobox genes in hematopoietic cells

**Fig 1:**
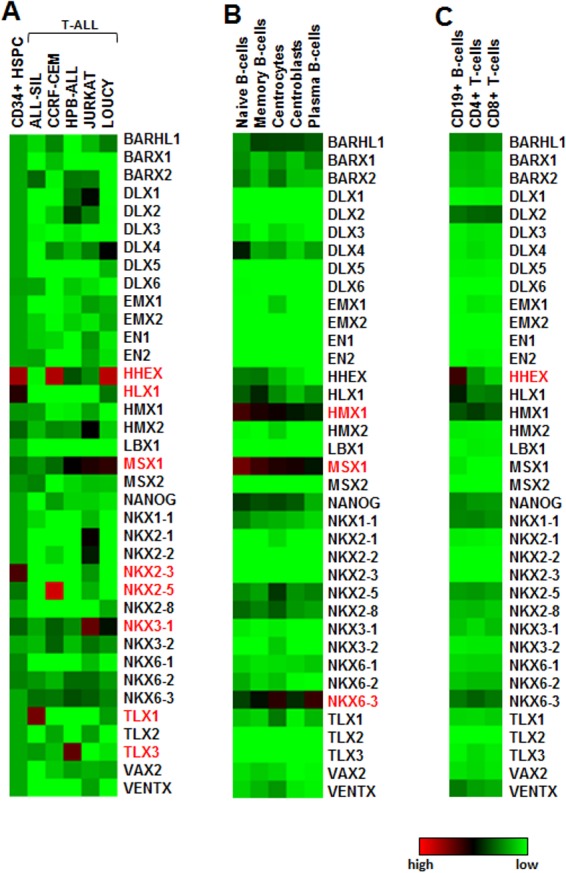
Gene expression profiling data of NKL homeobox genes in primary hematopoietic cells and cell lines. (A) Heatmap for primary HSPCs and 5 T-ALL cell lines. (B) Heatmap for primary immature B-cells. Data were obtained from GSE12453. (C) Heatmap for primary mature lymphocytes (GSE72642). Activated genes are indicated in red.

Expression profiling data obtained from primary HSPCs and selected T-ALL cell lines were illustrated as a heatmap in **Fig 1A**, showing transcript levels of 37 NKL homeobox genes. These data indicated that three genes, namely HHEX, HLX1 and NKX2-3, were physiologically expressed in HSPCs, while the analyzed T-ALL cell lines aberrantly expressed HHEX, MSX1, NKX2-5, NKX3-1, TLX1 and TLX3 as reported previously [8,10,15,16,31]. Thus, NKL homeobox oncogenes might either be hematopoietically reactivated (HHEX, MSX1) or ectopically activated (NKX2-5, TLX1, TLX3). Analysis of B-cell progenitors from peripheral blood and tonsils (dataset GSE12453) revealed physiological expression of another set of genes, comprising HMX1, MSX1 and NKX6-3 (**Fig 1B**). Alterations in expression levels indicated developmental downregulation of the genes HMX1 and MSX1 upon B-cell maturation, while NKX6-3 showed unsystematic variations. Analysis of differentiated lymphocytes (dataset GSE72642) indicated significant expression levels just of HHEX in mature B-cells, while mature T-cells showed absence of any NKL homeobox gene activity (**Fig 1C**). Thus, NKL homeobox genes display specific expression patterns which differ between particular stages during early hematopoiesis and lymphopoiesis indicating functional roles in development.

**Fig 2:**
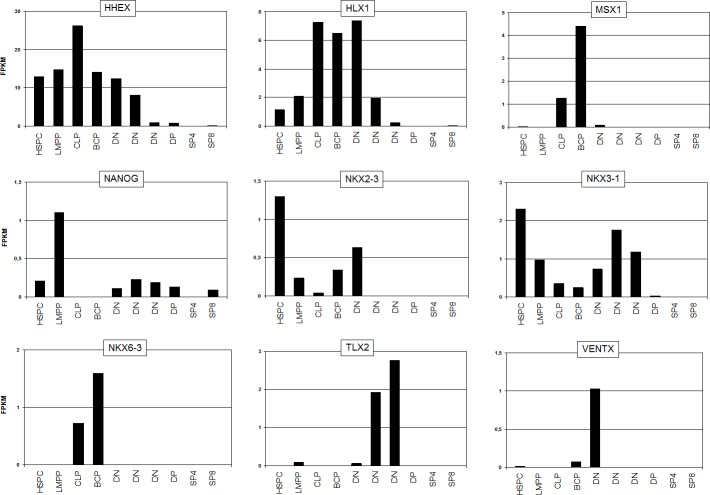
Expression of NKL homeobox genes in hematopoietis. RNA-sequencing data (GSE69239) were exploited to analyze all NKL homeobox genes in hematopoiesis. Nine genes were found to be expressed at significant levels which are indicated in FPKM (fragments per kilobase of mappable gene length and million reads). HSPC: hematopoietic stem and progenitor cells, LMPP: lymphoid and myeloid progenitor, CLP: common lymphoid progenitor, BCP: B-cell progenitor, DN: double negative T-cell progenitor, DP: double positive T-cell progenitor, SP4: single positive CD4+ T-cell progenitor, SP8: single positive CD8+ T-cell progenitor.

**Fig 3:**
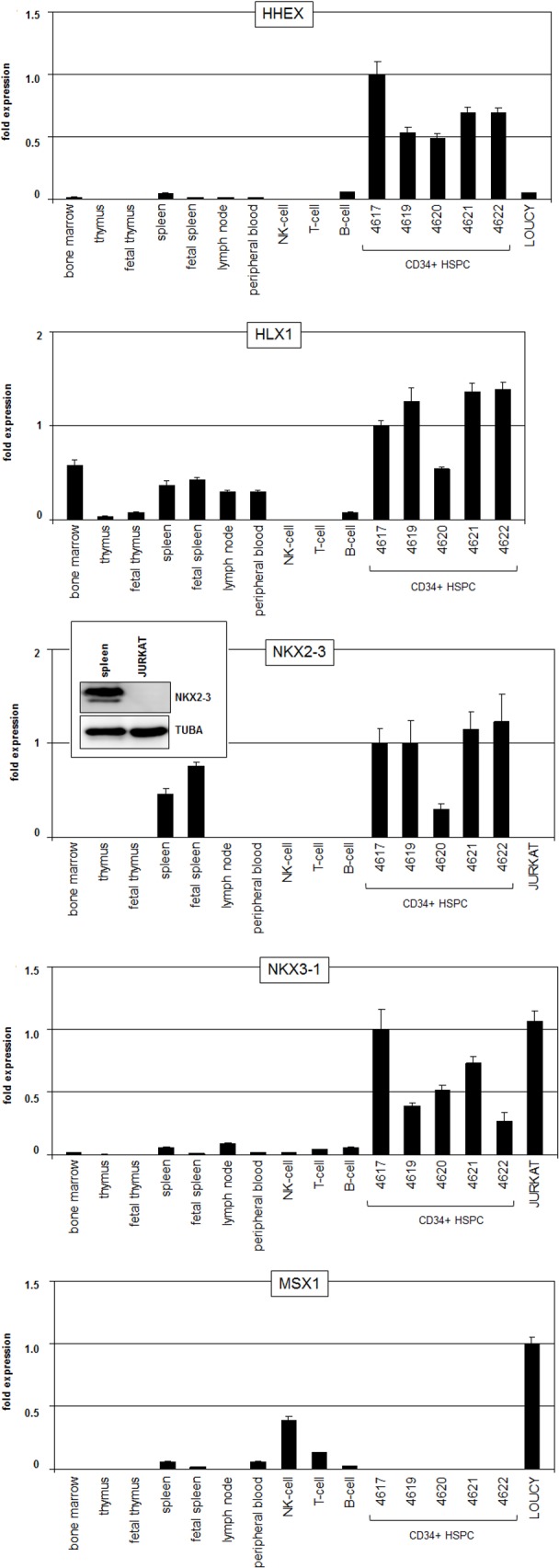
RQ-PCR expression analyses of NKL homeobox genes. Transcript levels of five selected NKL homeobox genes were quantified in primary hematopoietic cells/tissues and selected T-ALL cell lines. NKX2-3 expression was additionally analyzed by Western blot in spleen and JURKAT cells. Tubulin served as loading control.

Since this type of microarray profiling assay does not cover all 48 known NKL homeobox genes as listed by the HUGO Gene Nomenclature Committee, we analyzed for a comprehensive survey of the complete subclass of NKL homeobox genes public RNA-sequencing data (GSE69239) obtained from several hematopoietic entities (**Table 1**). These data demonstrated expression of four members in HSPCs comprising again HHEX, HLX1, NKX2-3 and additionally NKX3-1 (**Fig 2**). To validate these data we performed RQ-PCR quantifying these four NKL homeobox genes in addition to MSX1. We analyzed primary hematopoietic cells/tissues and selected T-ALL cell lines (**Fig 3**), supporting potent expression of HHEX, HLX1, NKX2-3 and NKX3-1 in HSPCs. Mature lymphocytes showed absent or faint expression levels of the genes analyzed. Furthermore, MSX1 was significantly expressed in NK-cells, indicating a functional role of MSX1 for this lineage of lymphocyte differentiation. HLX1 showed reduced expression in lymphatic tissues and NKX2-3 strong transcript levels in the spleen as described previously [19], while expression of HHEX and NKX3-1 was restricted to HSPCs. T-ALL cell lines LOUCY and JURKAT expressed HHEX, NKX3-1 and MSX1 as shown previously [14,15,17], and served as models for homeobox gene regulatory examinations (see below).

**Table 1:** NKL homeobox gene expressions in hematopoiesis (GSE69239) and T-ALL (GSE26713).

NKL gene	synonyms	HSC	LMPP	CLP	BCP	TCP (DN)	TCP (DP,SP)	T-ALL	chr. transl.
BARHL1									
BARHL2									
BARX1									
BARX2									
BSX	BSX1								
DBX1									
DBX2									
**DLX1**								**X**	
**DLX2**								**X**	
**DLX3**								**X**	
DLX4									
DLX5									
**DLX6**								**X**	
EMX1									
EMX2									
EN1									
EN2									
**HHEX**	PRHX	X	X	X	X	X		**X**	
**HLX**	HLX1, HB24	X	X	X	X	X		**X**	
HMX1	NKX5-3								
HMX2	NKX5-2								
HMX3	NKX5-1								
**LBX1**								**X**	
LBX2									
**MSX1**				X	X			**X**	
**MSX2**								**X**	
NANOG			X						
**NKX1-1**								**X**	
NKX1-2									
**NKX2-1**	TITF1, TTF1							**X**	X
**NKX2-2**								**X**	X
**NKX2-3**		X						**X**	
NKX2-4									
**NKX2-5**	CSX							**X**	X
NKX2-6									
NKX2-8									
**NKX3-1**	BAPX2	X				X		**X**	
**NKX3-2**	BAPX1							**X**	
NKX6-1									
NKX6-2									
**NKX6-3**					X			**X**	
NOTO									
**TLX1**	HOX11							**X**	X
**TLX2**	HOX11L1					X		**X**	
**TLX3**	HOX11L2							**X**	X
VAX1									
VAX2									
VENTX	VENTX2					X			
**48**		**4**	**3**	**3**	**4**	**5**	**0**	**20**	**5**

Significant transcript levels in the corresponding hematopoietic cell type are indicated by a cross and by grey background. Aberrantly overexpressed genes in T-ALL subsets are indicated by bold crosses. Genes involved in chromosomal aberrations in T-ALL are indicated by underlined crosses. HSPC: hematopoietic stem and progenitor cells, LMPP: lymphoid and myeloid progenitor, CLP: common lymphoid progenitor, BCP: B-cell progenitor, TCP(DN): T-cell progenitor (combined double negative T-cell progenitors), TCP(DP,SP): combined double positive and single positive T-cell progenitors.

Interestingly, while analyzing further hematopoietic entities of the RNA-sequencing dataset GSE69239, we identified the expression of additional NKL homeobox genes (**Table 1**, **Fig 2**). Lymphoid and myeloid progenitors (LMPP) expressed NANOG, common lymphoid progenitors (CLP) expressed HHEX, HLX1 and MSX1, B-cell progenitors (BCP) expressed HHEX, HLX1, MSX1 and NKX6-3, and progenitors representing early T-cell developmental DN stages expressed HHEX, HLX1, NKX3-1, TLX2 and VENTX at significant levels. Of note, late stages of T-cell development (DP, SP4 and SP8) do not express any NKL homeobox gene consistent with expression profiling data shown above (**Fig 1**). Overall, our analyses indicated that nine particular NKL homeobox genes are physiologically transcribed in HSPCs and lymphopoiesis, showing stage-specific expression patterns. Therefore, these results suggest the presence of an NKL-code for defined hematopoietic stages.

### Transcriptional regulation of HHEX and NKX3-1

**Fig 4:**
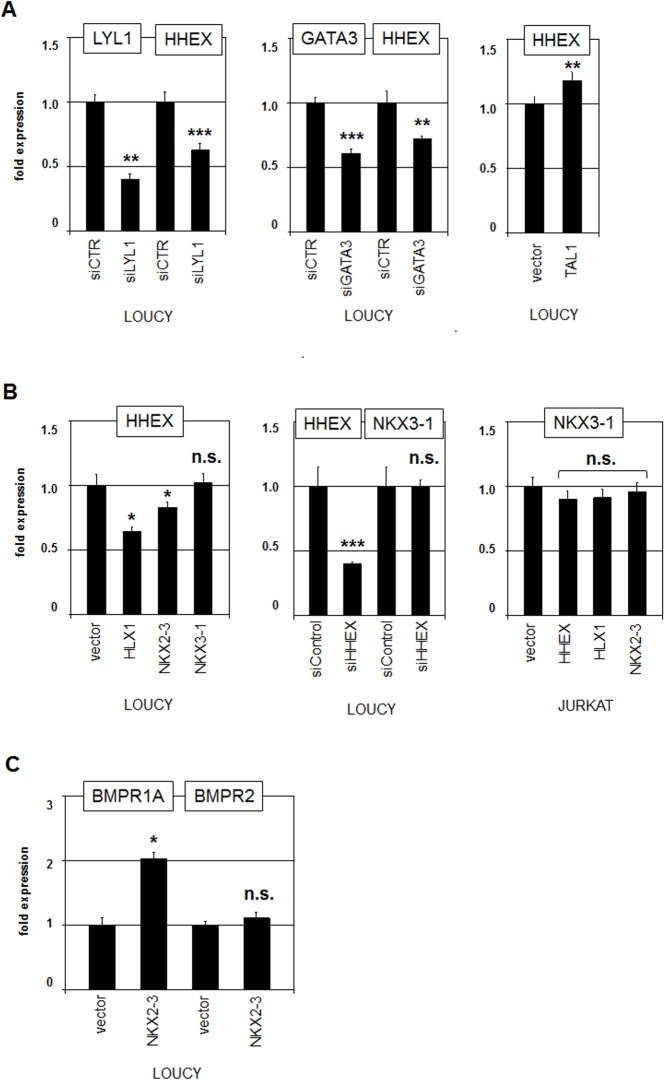
Analyses of NKL homeobox genes in T-ALL cell lines. (A) RQ-PCR analysis of HHEX after siRNA-mediated knockdown in LOUCY. Concomitant reduction of transcript levels indicated that LYL1 (left) and GATA3 (middle) activate the expression of HHEX. Forced expression of TAL1 resulted in slightly increased expression of HHEX (right), indicating an activating input as well. (B) RQ-PCR analysis of HHEX after forced expression of NKL homeobox genes HLX1, NKX2-3 and NKX3-1 in LOUCY (left). RQ-PCR analysis of NKX3-1 after siRNA-mediated knockdown of HHEX in LOUCY (middle). RQ-PCR analysis of NKX3-1 after forced expression of NKL homeobox genes HHEX, HLX1 and NKX2-3 in JURKAT (right). (C) RQ-PCR analysis of BMPR1A and BMPR2 after forced expression of NKL homeobox gene NKX2-3 in LOUCY.

To understand the formation of the described expression patterns in hematopoietic cells we analyzed regulation and capacities of selected NKL homeobox genes. As shown previously, the transcriptional regulation of NKX3-1 is governed by hematopoietic stem cell TFs TAL1 and LYL1 in addition to T-cell TF GATA3 which mediate its activation [14,15]. To analyze the regulation of HHEX in the same way we used here the immature T-ALL cell line LOUCY as model system. SiRNA-mediated knockdown of LYL1 or GATA3 was confirmed by RQ-PCR and resulted in concurrently reduced expression levels of HHEX (**Fig 4A**). Forced expression of TAL1 in LOUCY cells mediated slightly increased HHEX transcription indicating that overlapping TF complexes present in HSPCs regulate both HHEX and NKX3-1. Furthermore, forced expression of HLX1 or NKX2-3 in LOUCY cells resulted in decreased HHEX expression levels, while NKX3-1 overexpression mediated no alteration (**Fig 4B**). SiRNA-mediated knockdown of HHEX showed no alteration in NKX3-1 expression levels (**Fig 4B**), indicating absence of mutual regulation. Similarly, forced expression of HHEX, HLX1 or NKX2-3 in JURKAT cells showed no alterations of NKX3-1 expression (**Fig 4B**). Thus, while TFs of the basic helix-loop-helix and GATA families activate both NKX3-1 and HHEX, the expression of HHEX is suppressed by HLX1 and NKX2-3, together forming a regulatory network which contains NKL homeobox genes as basic mediators.

### Analysis of NKL homeobox gene NKX2-3

NKX2-3 represents a novel player of the NKL homeobox gene subclass in hematopoiesis. Western blot analysis of NKX2-3 was performed for spleen samples, demonstrating robust protein expression in this hematopoietic tissue (**Fig 3**). The NKX2-3 RNA expression levels in spleen were similar to those in HSPCs, suggesting comparable protein amounts of this factor in both cell types. Unfortunately, we were unable to find an NKX2-3 expressing T-cell line to analyze its transcriptional regulation. However, NKX2-3 expression is regulated by transcription factor NFATC2 as identified by single nucleotide polymorphism (SNP)-analyses of patients suffering inflammatory bowel disease [32]. High NFATC2 levels in HSPCs correlate with NKX2-3 (**S1 Fig**), suggesting a common regulatory relationship. But analysis of T-ALL cases overexpressing NKX2-3 did not show enhanced NFATC2 levels (GSE26713), excluding involvement of this factor in aberrant NKX2-3 regulation.

To identify potential target genes we compared expression profiling data of two NKX2-3 positive and ten NKX2-3 negative T-ALL patient samples (GSE26713, **S2 Fig**). For this approach we used the online tool GEO2R, revealing the 250 most significant differentially expressed genes (**S1 Table**), 50 of which showed decreased levels. Interestingly, one of the downregulated genes was NKL homeobox gene HHEX, which corresponded to our previous results, supporting that NKX2-3 repressed HHEX gene activity (**Fig 4B**). Gene-annotation enrichment analysis of the 200 upregulated genes highlighted activation of the BMP-signalling pathway (KEGG). Consistently, RQ-PCR analysis of LOUCY cells treated for forced expression of NKX2-3 demonstrated increased expression of the BMP receptor encoding gene BMPR1A (**Fig 4C**), supporting the notion that the BMP-signalling pathway is both located downstream and activated by NKX2-3.

### Regulation of MSX1 by stem cell NKL homeobox genes

**Fig 5:**
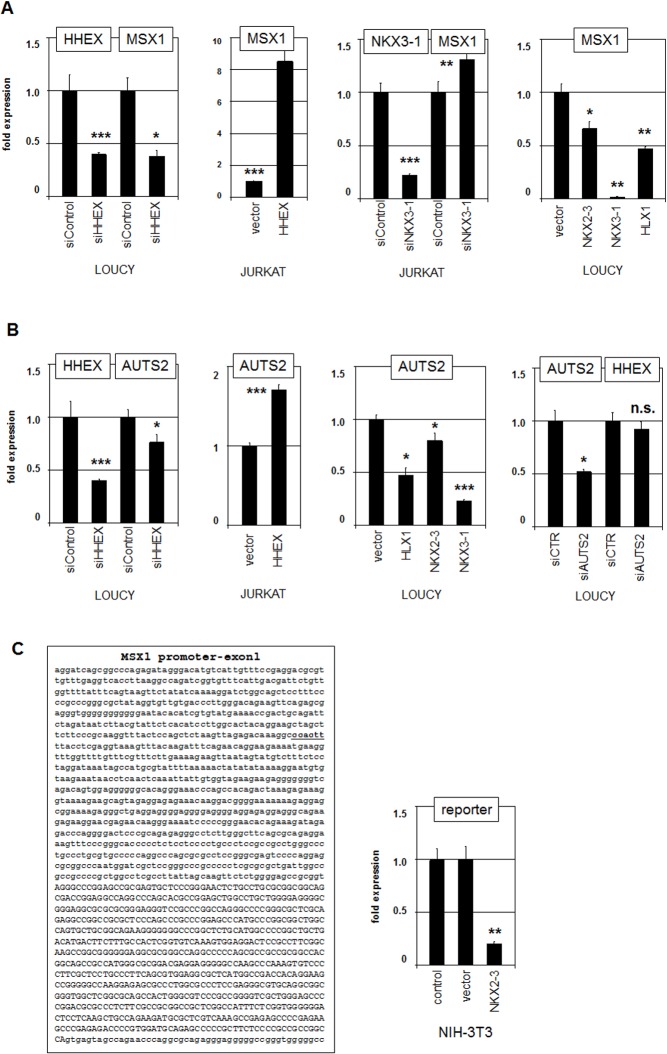
Analyses of MSX1 regulation in T-ALL cell lines. (A) RQ-PCR analysis of MSX1 after siRNA-mediated knockdown of HHEX in LOUCY and after forced expression of HHEX in JURKAT demonstrated that HHEX activates MSX1 transcription. RQ-PCR analysis of MSX1 after siRNA-mediated knockdown of NKX3-1 in JURKAT and after forced expression of NKX2-3, NKX3-1 and HLX1 in LOUCY demonstrated that all analyzed genes repressed MSX1 transcription. (B) RQ-PCR analysis of AUTS2 after siRNA-mediated knockdown of HHEX in LOUCY and after forced expression of HHEX in JURKAT demonstrated that HHEX activates AUTS2 transcription. RQ-PCR analysis of AUTS2 after forced expression of NKL homeobox genes HLX1, NKX2-3, NKX2-5 and NKX3-1 in LOUCY demonstrated that the analyzed homeobox genes inhibit transcription of AUTS2. RQ-PCR analysis of HHEX after siRNA-mediated knockdown of AUTS2 in LOUCY demonstrated that AUTS2 did not regulate HHEX expression. (C) The sequence shown comprises the regulatory upstream region and the first exon of MSX1 (capital letters). Reporter gene analysis was performed of a MSX1 promoter fragment containing an identified consensus NKX2-3 site (underlined). RQ-PCR analysis of the reporter gene showed that NKX2-3 directly represses MSX1 transcription (right).

Expression data for MSX1 showed that this NKL homeobox gene is turned off in HSPCs but activated in lymphoid progenitors (**Figs 2–3**). To analyze potential consequences of NKL factors expressed in HSPCs on MSX1 we performed knockdown and overexpression assays in MSX1-positive T-ALL cell lines. SiRNA-mediated knockdown of HHEX in LOUCY resulted in reduced expression of MSX1 (**Fig 5A**). Accordingly, forced expression of HHEX in JURKAT resulted in elevated MSX1 transcript levels, demonstrating that HHEX mediates activation of MSX1 expression (**Fig 5A**). SiRNA-mediated knockdown of NKX3-1 in JURKAT resulted in elevated expression of MSX1 (**Fig 5A**). Accordingly, forced expression of HLX1, NKX2-3 or NKX3-1 resulted in suppressed MSX1 transcription (**Fig 5A**). Thus, while HHEX activated MSX1 transcription, NKX2-3, NKX3-1 and HLX1 acted repressively, showing functional differences between NKL factors expressed in HSPCs and lymphoid progenitors, thus contributing to the observed NKL expression pattern.

Recently, we have shown that MSX1 transcription is regulated by the chromatin modulators AUTS2-polycomb repressor complex (PRC) 1 in T-ALL cells [17]. Therefore, we analyzed if the observed transcriptional impact of the NKL-factors on MSX1 operates via AUTS2. SiRNA-mediated knockdown of HHEX in LOUCY resulted in decreased AUTS2 expression, while its forced expression in JURKAT had the opposite effect (**Fig 5B**). In contrast, forced expression of HLX1, NKX2-3 or NKX3-1 resulted in reduced AUTS2 expression levels (**Fig 5B**). Thus, HHEX activated while HLX1, NKX2-3 and NKX3-1 repressed AUTS2 which in turn mediates the expression of MSX1. Of note, siRNA-mediated knockdown of AUTS2 left the expression level of HHEX unperturbed (**Fig 5B**), discounting regulation of this NKL homeobox gene via the AUTS2 chromatin complex.

Sequence analysis of the promoter region of MSX1 revealed one potential binding site for NKX2-3 at –656 bp [33]. To test if this NKL factor mediates direct regulation of MSX1 transcription we performed reporter gene assay for a corresponding fragment of the regulatory region (**Fig 5C**). The results showed that NKX2-3 repressed the reporter gene activity, demonstrating direct inhibitory regulation. Thus, MSX1 transcription is regulated by particular NKL homeodomain factors acting directly and/or indirectly by chromatin modulation via AUTS2.

### Deregulated NKL homeobox genes in T-ALL

Visual analyses of 37 NKL homeobox gene expression profiles for 117 T-ALL patient samples (GSE26713) confirmed enhanced transcript levels of NKL homeobox oncogenes including TLX1, TLX3, NKX2-1 and NKX2-5 and additionally identified novel members showing aberrant expression levels in patient subsets including DLX1, NKX2-3 and TLX2 (**S2 Fig**). These results were combined with expression data obtained from normal HSPCs, lymphoid progenitors and T-cell progenitors as indicated above (**Table 1**). According to this procedure we identified 20 deregulated genes, seven of which (35%) are physiologically expressed during hematopoiesis/lymphopoiesis (HHEX, HLX1, MSX1, NKX2-3, NKX3-1, NKX6-3, TLX2), while 13/20 (65%) are ectopically expressed in T-ALL (DLX1, DLX2, DLX3, DLX6, LBX1, MSX2, NKX1-1, NKX2-1, NKX2-2, NKX2-5, NKX3-2, TLX1, TLX3). Thus, ectopic activation represents the most frequent mode of deregulation, although reactivation (NKX2-3, NKX6-3) and failed downregulation (HHEX, HLX1, MSX1, NKX3-1, TLX2) play significant roles as well. Interestingly, chromosomal translocations are exclusively involved in ectopic activation of NKL homeobox genes (NKX2-1, NKX2-2, NKX2-5, TLX1, TLX3), suggesting the requirement of particular activating mechanisms for genes normally silenced in hematopoietic cells.

To look for coinciding gene activities of T-ALL cases expressing NKL homeobox genes we performed bioinformatic analyses of the T-ALL patient data set GSE26713. For this approach, the patient cohort was divided into two groups: 93 NKL-positive and 24 NKL-negative controls. The adopted online tool GEO2R extracted 250 differentially expressed genes according to their statistical significance (**S2 Table**). Subsequent gene-annotation enrichment analysis of these genes indicated activation of survival pathway (KEGG) and inhibition of FAS signalling pathway activity (BIOCARTA), highlighting deregulation of apoptosis (data not shown). Furthermore, we performed comparative expression profiling of five NKL-positive and five NKL-negative T-ALL cell lines (**S3 Table**). Gene-annotation enrichment analysis of the top 1000 differentially expressed genes highlighted various signalling pathways, including activation of MAPK- and TCR-pathways (KEGG, not shown). Of note, common downstream effects of these pathways mediate the regulation of apoptosis in T-cell development [34–36].

Principal component analysis and clustering of the patient and cell line data did not reflect the classification of the NKL-positive/negative groups (**S3 Fig**), demonstrating absence of any common gene signature for NKL homeobox gene expressing samples. Moreover, statistical analyses of genes differentially expressed between these groups in patients and cell lines revealed absence of common gene activities (with p<0.5), again showing no significant overlap within NKL-positive or NKL-negative sample groups (not shown). Thus, both data sets indicated absence of any NKL-specific gene signature but rather promotion of cell survival activity which might represent the consensual character of NKL-positive T-ALLs.

## Discussion

Here, we performed NKL homeobox gene expression analyses in hematopoiesis, lymphopoiesis, T-cell development and T-cell leukemia. We detected four subclass members in HSPCs (HHEX, HLX1, NKX2-3, NKX3-1), three in CLPs (HHEX, HLX1, MSX1), four in BCPs (HHEX, HLX1, MSX1, NKX6-3), and five in DN early thymocytes (HHEX, HLX1, NKX3-1, TLX2, VENTX). No NKL homeobox gene expression was detected in late thymocytes or differentiated T-lymphocytes while at least MSX1 was expressed in differentiated NK-cells. Thus, these genes show stage-specific expression patterns which we propose to term hematopoietic “NKL-code”.

Homeobox genes control fundamental decisions during embryogenesis of the body axis, head and pharyngeal region, and hematopoietic system. Accordingly, homeobox gene codes have been described for these embryonal regions/tissues. The HOX-code was the first gene code recognized and describes the colinear expression pattern of clustered homeobox genes along the anterior-posterior axis in the head, the branchial region, and the vertebrae [21,37,38]. In addition, the branchial archs display the DLX-code mediating their dorso-ventral differentiation [22,23]. Within the pharyngeal region an NKX-code has been described, however, consisting of just three genes [39]. The hematopoietic system shows a specific expression pattern of the clustered homeobox genes which has been also termed HOX-code [40–42]. In particular, HOX members display specific expression patterns during T-cell differentiation, highlighting their developmental importance in lymphopoiesis [43]. Interestingly, deregulated HOX genes play key roles in T-ALL subsets [24,44], demonstrating a relationship between aberrant homeobox gene expression patterns and malignancy as shown here for the NKL subclass.

Transcripts of NKL homeobox genes HHEX, HLX1 and NKX2-3 but not of NKX3-1 have also been identified in embryonic HSPCs [45]. Of note, these data indicate a prominent role for NKX2-3 in early embryonic hematopoiesis. Furthermore, NKX2-3 is expressed during myelopoiesis and behaves as a tumor suppressor gene in acute myeloid leukemia subtypes [46]. In marginal zone B-cell lymphoma NKX2-3 is aberrantly activated and acts as an oncogene [47]. Our results indicate an oncogenic role of NKX2-3 in T-ALL as well.

**Fig 6:**
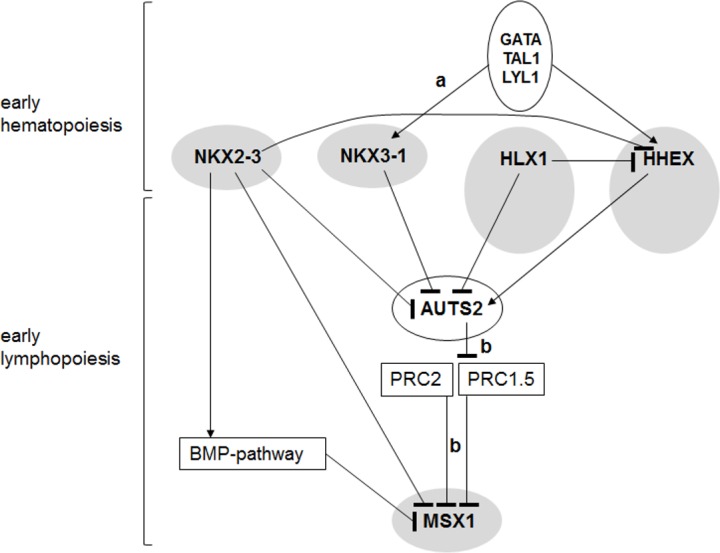
Gene regulatory network of hematopoietic NKL homeobox genes expressed in HSPCs and CLP. This figure summarizes the results obtained in this study in addition to published data: a [14,15], b [17]. NKL homeobox genes are highlighted with grey ovals indicating their expression in early hematopoiesis (NKX2-3, NKX3-1, HLX1, HHEX) and early lymphopoiesis (HLX1, HHEX, MSX1). Gene regulatory connections are given by arrows (activating) and blunt arrows (suppressing).

We used NKL homeobox gene expressing T-cell lines as models and revealed a regulatory network which might also be paralleled in hematopoietic stem and lymphoid progenitor cells (**Fig 6**). Accordingly, MSX1 is regulated by NKX2-3, AUTS2 and BMP-signalling, forming a marker gene for the human CLP as shown previously in the mouse [48]. In intestinal microvascular endothelial cells NKX2-3 enhances BMP-signalling via activation of BMP4 and inhibition of SMAD7 [49]. We have shown recently that the BMP-pathway inhibits MSX1 expression in T-ALL which is activated via aberrant suppression of BMP-signalling [16]. Here, we have shown that NKX2-3 inhibits MSX1 transcription directly, activates BMP-receptor expression, and inhibits AUTS2 expression, together contributing to MSX1 downregulation. In colon cancer cell lines NKX2-3 regulates AUTS2 as well [50], suggesting a direct regulatory connection. AUTS2 interacts with and turns polycomb repressor complex (PRC) 1 into an activating complex which is involved in MSX1 deregulation [17,51]. Consistent with this idea, expression data of AUTS2 and its PRC1 binding partner PCGF5 show elevated and reduced levels, respectively, in both CLPs and BCPs, corresponding to enhanced MSX1 transcript levels in these hematopoietic entities (**S1 Fig**). Furthermore, HHEX activated, while HLX1 and NKX3-1 repressed AUTS2 expression, displaying a regulatory network.

The expression of NKL homeobox gene HHEX is important for normal hematopoiesis [52]. The activity of HHEX is downregulated in T-cell differentiation while its overexpression mediates T-cell malignancy [53,54]. Overexpression of MSX1 has been described in T-ALL subsets as well [16]. Thus, both genes represent paradigms for aberrant activity of hematopoietic NKL homeobox genes. Our data indicate that aberrant chromosomal rearrangements mediate exclusively ectopic NKL homeobox gene expression in T-ALL. This observation might suggest that non-hematopoietic genes require T-cell specific enhancers for their activity in these cells which is realized by juxtaposition to TCR loci or BCL11B. However, in marginal-zone B-cell lymphoma NKX2-3 expression is aberrantly activated via chromosomal juxtaposition to the B-cell receptor gene, and in diffuse large B-cell lymphoma activation of NKX2-1 is mediated by chromatin alterations but not via direct chromosomal rearrangement [47,55]. These cases oppose the situation in T-ALL, suggesting lineage-dependent differences in gene regulation during B- and T-cell development.

Furthermore, the expression levels of NKL homeo-oncogenes might themselves be of significance. High HHEX mediates suppression of hematopoietic differentiation, while low levels mediate development of T-cell malignancies [54]. This interrelation has been described for aberrantly expressed NKX2-5 and NKX3-1 as well [15,55]. Moreover, the presence of an NKL-code in lymphoid progenitors and the absence of NKL homeobox gene activity in mature T-cells support the sensitivity of these progenitor cell types for transformation by this gene class. However, deregulated NKL homeobox genes have been described in B-cell malignancies as well [47,55]. But this oncogene class seems to be much less prevalent in those types of lymphoid tumours.

Our data indicated absence of common target genes of NKL homeodomain proteins in T-ALL, although several target genes have been described for TLX1, TLX3 and NKX2-5 in this malignancy [56–59]. However, these results may reflect differences in DNA-binding site specificities as shown for MSX1, NKX3-1 and NKX2-1 [60]. Furthermore, NKL homeodomain proteins share a conserved segment in their N-terminal regions. This EH1 domain mediates protein-interactions with TLE/groucho corepressors, and might be profoundly important for their leukemic impact masking differences in DNA-binding specificity [61–63]. The basic role of NKL homeobox genes and their TLE corepressors has been highlighted in the embryonal development of the ventral neural tube [64], suggesting that repression of alternative differentiation programs represents a general function of these genes.

## Supporting information

S1 Fig Expression levels of selected genes in hematopoietic cell types (GSE69239). (TIF)

S2 Fig **(A) NKL homeobox gene expression levels in 117 T-ALL patients (GSE26713).** Bars indicate expression levels of the indicated genes. The selected genes show aberrantly elevated levels in subsets of the patients. The first seven samples correspond to bone marrow controls of healthy donors. The selected genes include DLX genes. (**B) NKL homeobox gene expression levels in 117 T-ALL patients (GSE26713).** Bars indicate expression levels of the indicated genes. The selected genes show aberrantly elevated levels in subsets of the patients. The first seven samples correspond to bone marrow controls of healthy donors. The selected genes include NKX genes. (**C) NKL homeobox gene expression levels in 117 T-ALL patients (GSE26713).** Bars indicate expression levels of the indicated genes. The selected genes show aberrantly elevated levels in subsets of the patients. The first seven samples correspond to bone marrow controls of healthy donors. The selected genes include TLX genes.
(TIF)

S3 Fig Principal component analysis and cluster analysis of T-ALL (A) patients and (B) cell lines. Of note, the patient data were obtained from GS26713 and include seven bone marrow samples from healthy donors which were named here controls.
(TIF)

S1 Table GEO2R analysis of NKX2-3 positive/negative T-ALLs patients (GSE26713). NKX2-3 positive samples: GSM657744, GSM657745. NKX2-3 negative controls: GSM657733, GSM657734, GSM657735, GSM657736, GSM657737, GSM657738, GSM657739, GSM657740, GSM657741, GSM657742.
(XLSX)

S2 Table GEO2R analysis of NKL-positive/negative T-ALL patients (GSE26713). NKL positive samples: GSM657722, GSM657723, GSM657726, GSM657727, GSM657729, GSM657731, GSM657732, GSM657733, GSM657734, GSM657735, GSM657736, GSM657737, GSM657738, GSM657739, GSM657740, GSM657741, GSM657742, GSM657743, GSM657744, GSM657745, GSM657746, GSM657747, GSM657749, GSM657750, GSM657753, GSM657754, GSM657755, GSM657756, GSM657757, GSM657758, GSM657759, GSM657760, GSM657761, GSM657762, GSM657765, GSM657766, GSM657767, GSM657768, GSM657769, GSM657770, GSM657771, GSM657773, GSM657775, GSM657776, GSM657777, GSM657778, GSM657779, GSM657780, GSM657781, GSM657782, GSM657783, GSM657784, GSM657785, GSM657786, GSM657787, GSM657788, GSM657789, GSM657793, GSM657794, GSM657795, GSM657796, GSM657797, GSM657798, GSM657799, GSM657801, GSM657802, GSM657804, GSM657805, GSM657806, GSM657807, GSM657808, GSM657809, GSM657810, GSM657814, GSM657815, GSM657816, GSM657817, GSM657821, GSM657822, GSM657823, GSM657824, GSM657825, GSM657826, GSM657827, GSM657828, GSM657829, GSM657830, GSM657831, GSM657833, GSM657834, GSM657835, GSM657836, GSM657837. NKL negative samples: GSM657721, GSM657724, GSM657725, GSM657728, GSM657730, GSM657748, GSM657751, GSM657752, GSM657763, GSM657764, GSM657772, GSM657774, GSM657790, GSM657791, GSM657792, GSM657800, GSM657803, GSM657811, GSM657812, GSM657813, GSM657818, GSM657819, GSM657820, GSM657832.
(XLSX)

S3 Table Differential gene expression analysis of NKL-positive (ALL-SIL, CCRF-CEM, HPB-ALL, LOUCY, PEER) and negative T-ALL cell lines (CML-T1, MOLT-4, MOLT-14, MOLT-16, SUP-T1) (GSE87334). (XLSX)
