# Supplementary figures and images for: NKL homeobox gene activities in hematopoietic stem cells, T-cell development and T-cell leukemia
Source: PLoS One. 2017 Feb 2;12(2):e0171164. doi: 10.1371/journal.pone.0171164 (PMC5289504; doi:10.1371/journal.pone.0171164)

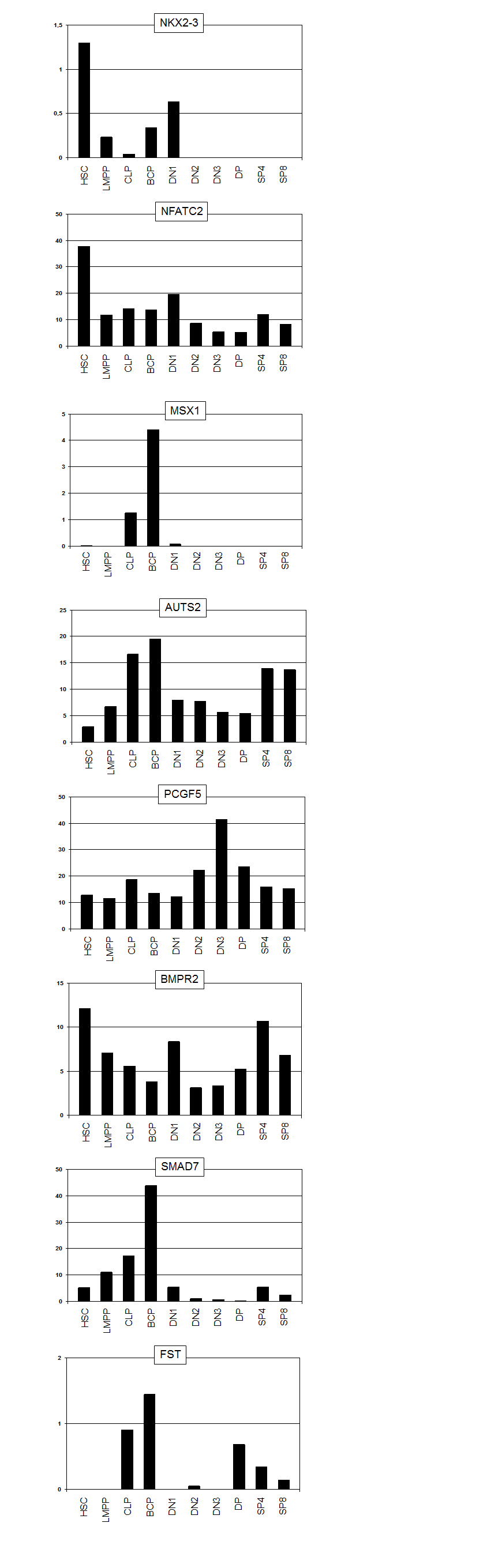

Supplement: S1 Fig — (TIF) [file pone.0171164.s001.tif]

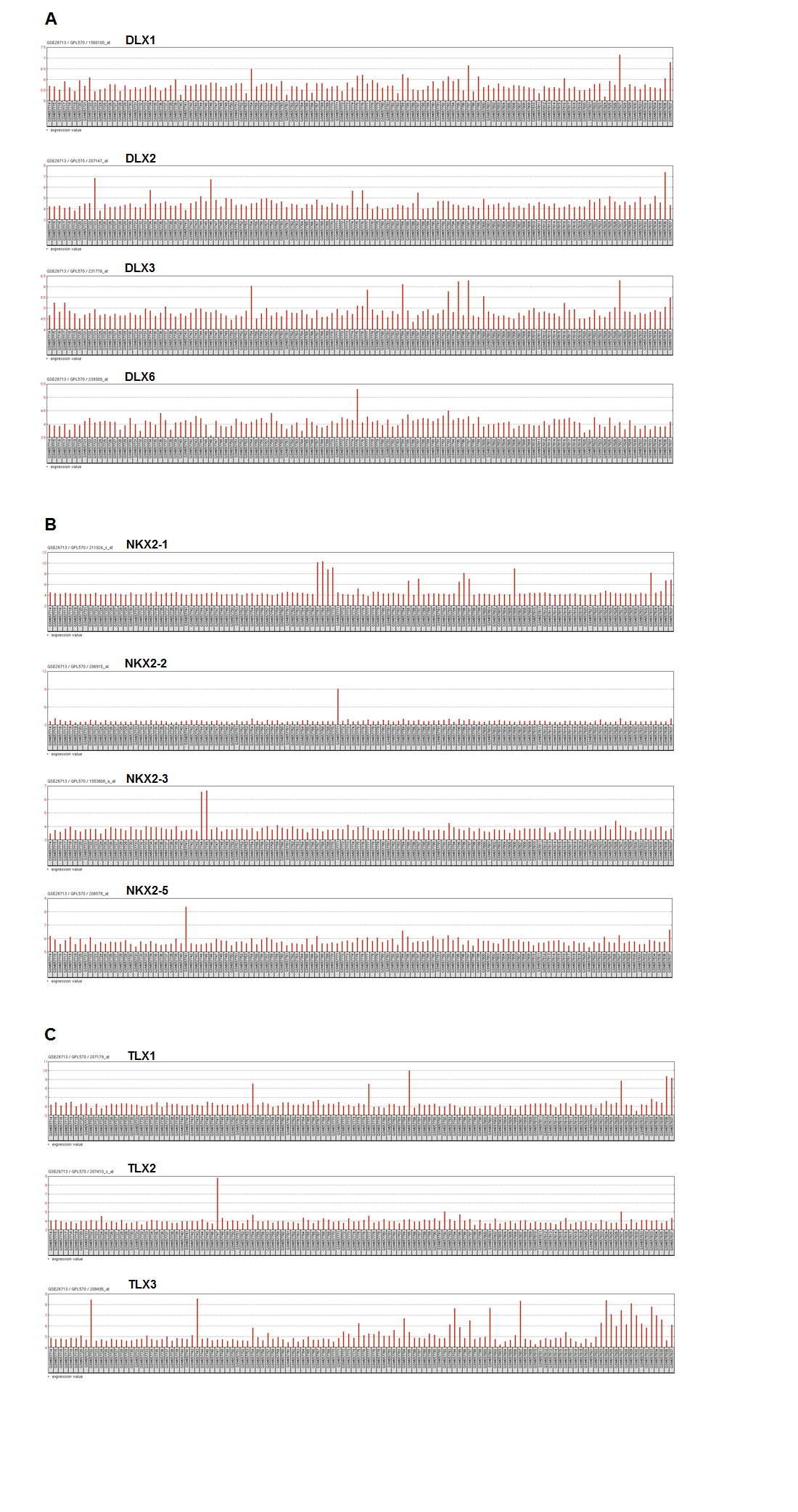

Supplement: S2 Fig — (A) NKL homeobox gene expression levels in 117 T-ALL patients (GSE26713). Bars indicate expression levels of the indicated genes. The selected genes show aberrantly elevated levels in subsets of the patients. The first seven samples correspond to bone marrow controls of healthy donors. The selected genes include DLX genes. (B) NKL homeobox gene expression levels in 117 T-ALL patients (GSE26713). Bars indicate expression levels of the indicated genes. The selected genes show aberrantly elevated levels in subsets of the patients. The first seven samples correspond to bone marrow controls of healthy donors. The selected genes include NKX genes. (C) NKL homeobox gene expression levels in 117 T-ALL patients (GSE26713). Bars indicate expression levels of the indicated genes. The selected genes show aberrantly elevated levels in subsets of the patients. The first seven samples correspond to bone marrow controls of healthy donors. The selected genes include TLX genes. (TIF) [file pone.0171164.s002.tif]

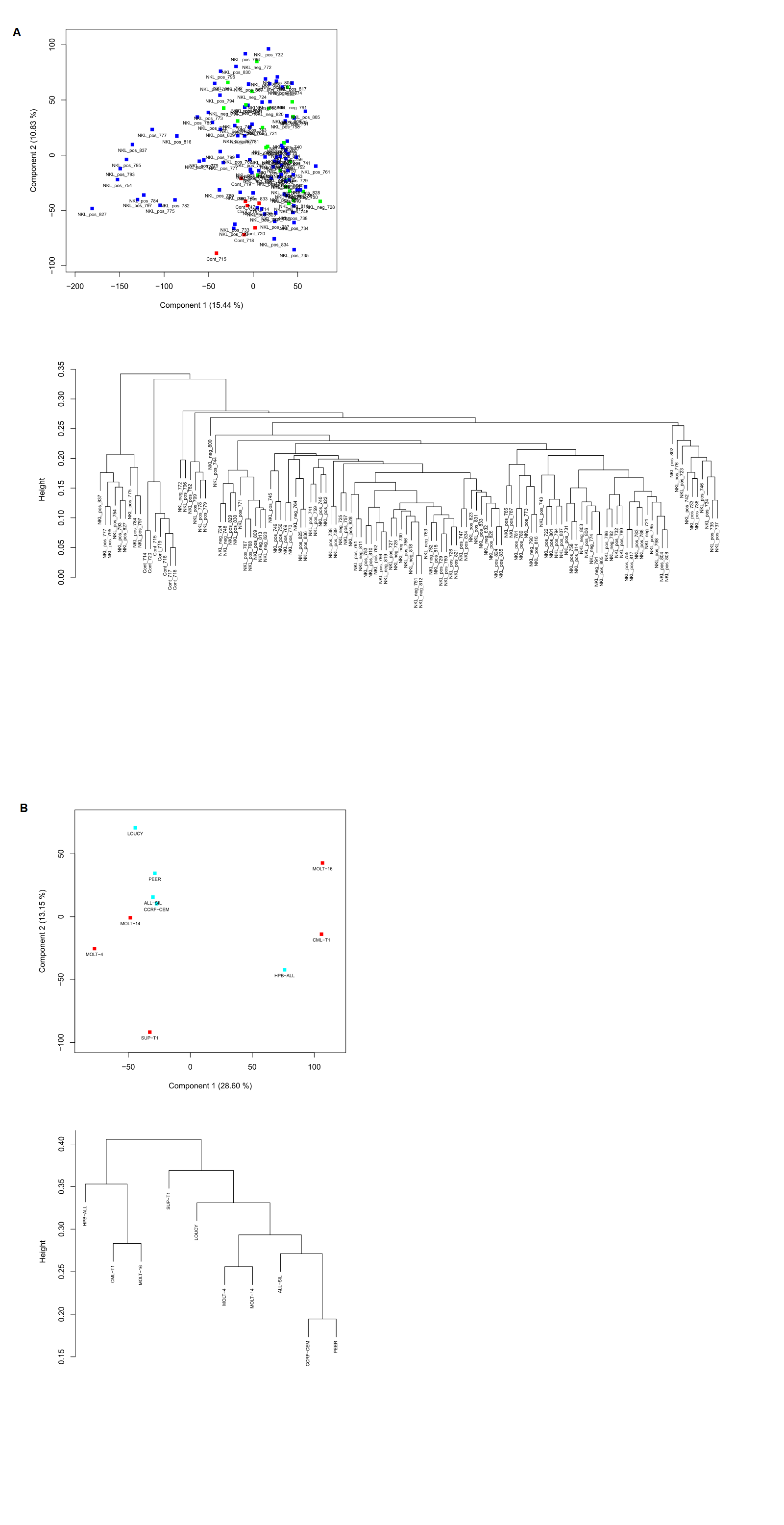

Supplement: S3 Fig — Principal component analysis and cluster analysis of T-ALL (A) patients and (B) cell lines. Of note, the patient data were obtained from GS26713 and include seven bone marrow samples from healthy donors which were named here controls. (TIF) [file pone.0171164.s003.tif]
